# β-trace protein is highly removed during haemodialysis with high-flux and super high-flux membranes

**DOI:** 10.1186/s12882-017-0489-6

**Published:** 2017-02-20

**Authors:** Carlo Donadio, Danika Tognotti, Laura Caponi, Aldo Paolicchi

**Affiliations:** 1 Division of Nephrology, Department of Clinical and Experimental Medicine, University of Pisa, Via Savi 10, I-56126 Pisa, Italy; 2 Laboratory of Clinical Pathology, Department of Translational Research, University of Pisa, Pisa, Italy

**Keywords:** β-trace protein, Low-molecular weight proteins, Maintenance haemodialysis, Permeability of dialysis membranes, Residual renal function

## Abstract

**Background:**

Serum β-trace protein (βTP, MW 23–29 kDa) is a marker of GFR impairment in renal patients. Recent papers propose to predict residual renal function (RRF) in maintenance haemodialysis (MHD) patients from serum concentrations of βTP and other small proteins, avoiding the collection of urine. Few data are available on the removal of βTP in patients treated with dialysis membranes with different flux characteristics. The aim of this study was to evaluate the effects of haemodialysis with low-flux, high-flux and super high-flux membranes on serum concentrations of ßTP in MHD patients with null RRF.

**Methods:**

Serum ßTP concentrations were measured before and after the first dialysis of the week in 51 MDH patients treated by low-flux (*n* = 24), high-flux (*n* = 17), or super high-flux (*n* = 10) membranes. The removal of β2-microglobulin (β2M, MW 11.8), cystatin C (Cys, MW 13.3), urea and creatinine was also analyzed.

**Results:**

Low-flux membranes did not remove βTP, β2M and Cys whose concentration increased at the end of dialysis. High-flux membrane removed more efficiently β2M and Cys than ßTP. Super high-flux membrane had the highest efficiency to remove ßTP: mean reduction ratio (RR) 53.4%, similar to β2M (59.5%), and Cys (62.0%).

**Conclusions:**

In conclusion, the plasma clearance of small proteins and particularly of βTP is dependent from the permeability of the dialysis membranes Therefore, the reliability of the formulas proposed to predict RRF from serum βTP and other LMWP may be affected by the different permeability of the dialysis membranes.

## Background

The measurement of residual renal function (RRF) is clinically relevant in the management of maintenance haemodialysis (MHD) patients, since RRF significantly influences the adequacy of dialytic treatment and the survival of MHD patients [1–3]. A careful monitoring of RRF is particularly relevant when an incremental approach to the initiation of haemodialysis is used [4]. Frequently the evaluation of RRF is obtained from the measurement of urinary clearance of creatinine and urea, collecting the urine from the end of a dialysis to the beginning of the next dialysis and measuring at the same times the serum concentrations of creatinine and urea [5, 6].

β-trace protein (βTP), also known as lipocalin-type prostaglandin D synthase, is a small protein (molecular weight 23–29 kDa, depending on the different glycosylation of the molecule), isolated primarily from cerebrospinal fluid [7–10]. Like other low-molecular weight proteins (LMWPs), βTP is taken up by tubular cells and actively degraded within their lysosomes [4]. Studies in chronic kidney disease (CKD) patients demonstrated that serum βTP is an adequate marker of glomerular filtration rate (GFR) impairment with a diagnostic accuracy similar to those of serum creatinine, cystatin C (Cys) and β2-microglobulin (β2M) [11–15].

Quite recently the possibility to predict RRF in MHD patients from serum concentration of βTP combined with β2M, or with β2M and Cys, has been addressed [16, 17]. The need for studies comparing βTP clearance with high-flux hemodialysis, superflux dialyzers, and high-volume hemodiafiltration, has been highlighted [18]. In fact, the different efficiency of membranes in the removal of βTP from blood could affect the accuracy of formulas proposed to calculate RRF in MHD patients from serum βTP.

The aim of this study was to evaluate the effects of haemodialysis with low-flux, high-flux and super high-flux membranes on serum concentrations of ßTP in MHD patients with null RRF.

## Methods

We report the data on serum concentrations of ßTP in 51 MHD patients treated by low flux (*n* = 24), high-flux (*n* = 17), and super high-flux (*n* = 10) membranes. The removal from blood of β2M (MW 11.8), Cys (MW 13.3), urea and creatinine was also analyzed for comparison. These data, unpublished up to now, are from our database of studies on the dialytic efficiency of haemodialysis membranes. Patients were randomly allocated into the different treatment groups.

### Study design

Single center cross sectional study of prevalent MHD patients. Setting: haemodialysis facility of the Nephrology Division, Dept Medicine, University of Pisa.

Inclusion criteria: age >18 years; dialytic vintage >6 months; residual diuresis null.

Exclusion criteria: incapacity to give informed consent.

Blood samples were drawn before starting and after the end of the first haemodialysis of the week. To minimize a post-dialysis rebound phenomenon, blood samples were drawn 30 min after the end of dialysis. Serum samples were stored into Eppendorf tubes at −20 ° C up to biochemical determinations.

### Ethics, consent and permissions

The Institutional Ethical Committee Azienda Ospedaliero-Universitaria Pisana (2395/2007) approved the study on the dialytic efficiency of haemodialysis membranes, that was conducted according to Helsinki declarations. Patients gave verbally their informed consent.

### Dialyzers and membranes

Low-flux dialyzers: Polysulfone (F8, Fresenius, Bad Homburg, Germany); Cellulose diacetate (Acepal 1700, Diacepal 16, Hospal, Mirandola, Italy); High-flux dialyzer: Acrylonitrile and sodium methallyl sulfonate copolymer (Nephral 500, Hospal Gambro, Mirandola, Italy); Super high-flux dialyzer: Cellulose triacetate (N190 FH, Nipro, Japan). Main characteristics of the different dialyzers are reported in Table 1.

**Table 1:** Main characteristics and properties of the different dialyzers, according to manufacturers data

Permeability	Low-flux	Low-flux	High-flux	Super high-flux
Dialysis Membrane	Polysulfone	Cellulose diacetate	Acrylonitrile and sodium methallyl sulfonate copolymer	Cellulose triacetate
KUF, mL/h/mmHg	7.5	13–13.7	65	84.7
Surface, m^2^	1.8	1.6–1.7	2.15	1.9
Urea clearance, mL/min	186	183–190	195	199

KUF = ultrafiltration coefficient; Standard conditions: QB = 200 mL/min; QD = 500 mL/min; TMP 100 mmHg; QUF = 0–10 ml/min

### Laboratory methods

Urea was determined by an enzymatic method (UREA/BUN Roche/Cobas; Roche Diagnostics, Mannheim, Germany). Creatinine was measured with a rate-blanked creatinine/Jaffé method (CREA Roche/Hitachi automated analysis for Hitachi 917, Roche Diagnostics, Mannheim, Germany). β2M was measured with an immune-enzymic method (AxSym ß2-Microglobulin, Abbott, Wiesbaden, Germany; mean reference value 0.99 ± 0.16 mg/L). Cys was measured with a particle enhanced immune-nephelometric method (N Latex Cystatin C, Siemens Healthcare, Erlangen, Germany; reference intervals 0.53–0.95 mg/L). βTP was measured with a particle enhanced immune-nephelometric method (N Antiserum to human βTP, Siemens Healthcare, Erlangen, Germany). Reference intervals, calculated in our laboratory, were 0.37–0.77 mg/L in men, and 0.40–0.70 mg/L in women [14].

### Statistical analysis

Data are reported as means ± standard deviation. The significances of the differences between groups were assessed using non parametric Mann-Whitney test. Statistical analysis was performed using MedCalc Statistical Software version 16.4.3 (MedCalc Software, Ostend, Belgium). A *p* value <0.05 was considered statistically significant.

## Results

Anthropometric and clinical data of patients are reported in Table 2.

**Table 2:** Anthropometric and clinical data of the 51 patients

Haemodialysis membrane	Low-flux	High-flux	Super High-flux
Number (males)	24 (12)	17 (16)	10 (9)
Age, years	65.7 ± 19.7	59.2 ± 11.7	64.6 ± 15.3
Dialysis Vintage, years	4.4 ± 3.2	6.9 ± 4.2^x^	2.7 ± 1.9^**^
Body weight, kg	64.0 ± 20.2	73.3 ± 13.3	76.9 ± 20.9
Body height, cm	163 ± 12	169 ± 8.6	171 ± 15.3
BMI, kg/m^2^	23.6 ± 5.2	25.7 ± 4.5	25.4 ± 3.9
Native kidney disease			
Ischemic nephropathy	12 (50%)	2 (11.8%)	6 (60%)
Diabetic nephropathy	3 (12.5%)	2 (11.8%)	3 (30%)
Glomerulonephrites	3 (12.5%)	4 (23.5%)	1 (10%)
Interstitial nephrites	2 (8.3%)	4 (23.5%)	0
Chronic kidney disease	2 (8.3%)	3 (17.6%)	0
ADPKD and congenital nephropathies	2 (8.3%)	2 (11.8%)	0

Mean values and standard deviations, or numbers and percentages are reported. The statistical significance (*p*) of the differences between mean values are indicated as follows: High-flux vs low-flux: x *p* < 0.05; Super high-flux vs high-flux: ** *p* < 0.01

Few differences in dialytic prescription were found between the different groups of patients (Table 3).

**Table 3:** Dialysis parameters and serum concentrations of urea, creatinine, β-trace protein, β2-microglobulin and cystatin C before (BD) and after (AD) haemodialysis

	Low-Flux *n* = 24	High-Flux *n* = 17	Super High-Flux *n* = 10
	Mean ± SD	Mean ± SD	Mean ± SD
Dialysis length, h	3.7 ± 0.5	3.7 ± 0.3	4.2 ± 0.2^** §§^
Blood flow, mL/min	322 ± 40	368 ± 21^x^	330 ± 42
Dialysate flow, mL/min	500	500	500
Ultrafiltration, kg	2.8 ± 0.8	3.4 ± 0.9^x^	3.2 ± 1.0
Urea BD, mg/dL	145 ± 45	159 ± 40	122 ± 45
Urea AD, mg/dL	45 ± 20	48 ± 15	36 ± 18
Creatinine BD, mg/dL	9.5 ± 3.7	11.2 ± 1.9^x^	10.5 ± 3.5
Creatinine AD, mg/dL	3.7 ± 1.3	4.2 ± 0.9	3.9 ± 1.4
β-Trace protein BD, mg/L	11.8 ± 4.8	10.9 ± 2.2	8.11 ± 2.4^**§^
β-Trace protein AD, mg/L	14.8 ± 6.6	8.0 ± 2.5	3.8 ± 1.9^***§§§^
β2-Microglobulin BD, mg/L	39.4 ± 15.3	24.4 ± 3.6^xx^	29.0 ± 12.0
β2-Microglobulin AD, mg/L	42.9 ± 18.0	9.1 ± 1.4^xxx***^	11.4 ± 5.1^§§§^
Cystatin C BD, mg/L	7.4 ± 1.9	9.36 ± 2.6^xx^	5.29 ± 0.9^***§§^
Cystatin C AD, mg/L	7.4 ± 2.4	2.4 ± 0.6^xxx^	1.9 ± 0.8^§§§^

Mean values and standard deviations (SD) are reported. The statistical significance (*p*) of the differences between mean values are indicated as follows: High-flux vs low-flux: x *p* < 0.05; xx *p* < 0.01; xxx *p* < 0.001; Super high-flux vs low-flux: § *p* < 0.05; §§ *p* < 0.01; §§§ *p* < 0.001; Super high-flux vs high-flux: ** *p* < 0.01; *** *p* < 0.001

**Fig. 1:**
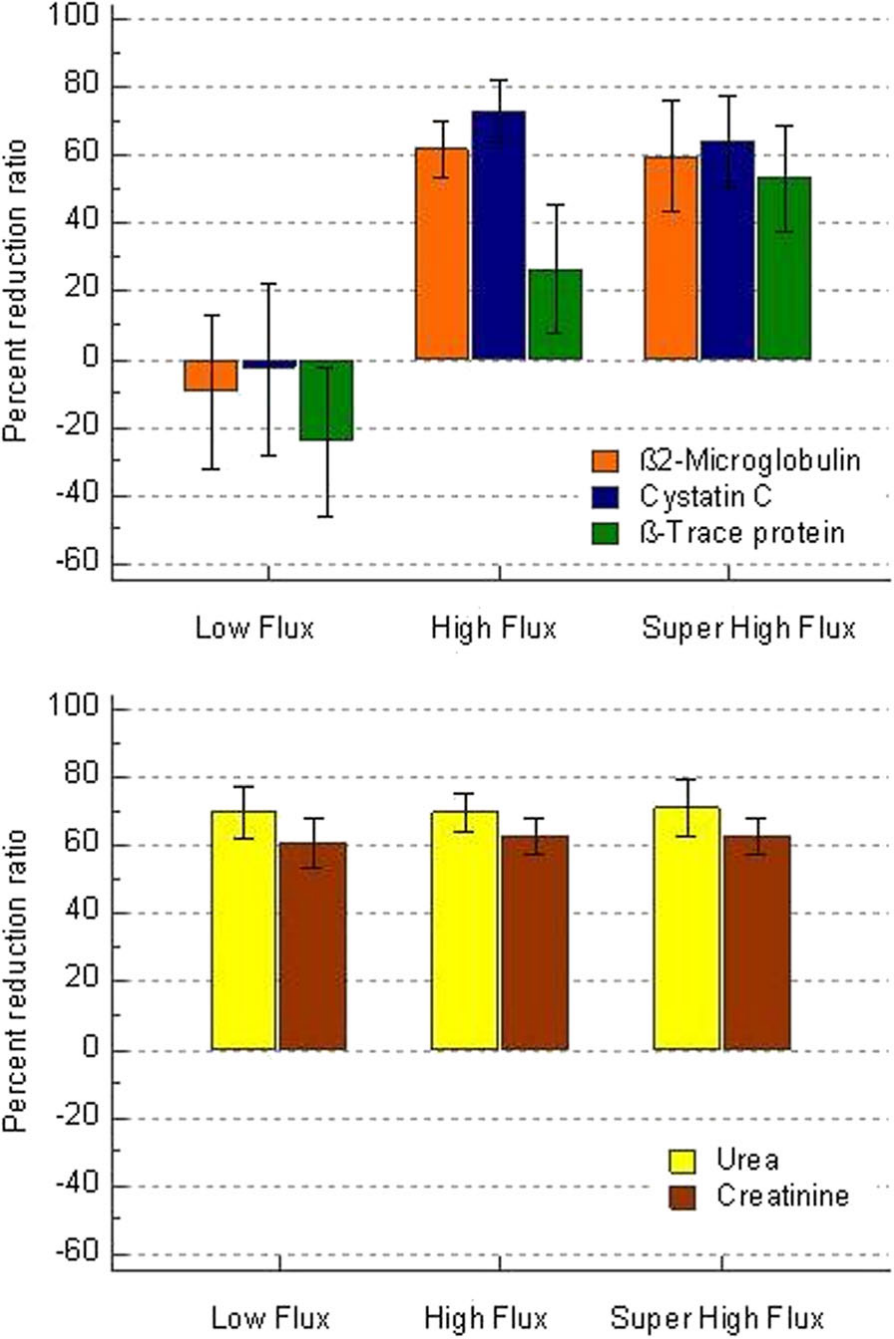
Percent reduction ratios of β2-microglobulin, cystatin C and β-trace protein in patients treated with dialyzers with different permeability: Low-flux, high-flux, and super high-flux. Percent reduction ratios of urea and creatinine of the same patients are reported for comparison. Mean values and 1 standard deviation from the mean are drawn

Serum βTP, β2M and Cys decreased significantly after the treatment with super high-flux and high-flux membranes. On the contrary, serum βTP significantly increased in patients treated with low-flux HD, due to the dialytic dehydration. Serum βTP levels increased to a similar extent after polysulfone or cellulose diacetate treatment: +27 ± 23 and +21 ± 22%, respectively (*p* = 0.50). In patients treated by super high-flux membrane, serum βTP was significantly lower, either before or after dialytic treatment, than in those treated with high-flux and low-flux membranes. The percent reduction ratios of β2-microglobulin, cystatin C and β-trace protein increased progressively according to the flux of the different membranes (Fig. 1).

In fact, low-flux membranes did not remove any LMWP. High-flux membrane removed more efficiently β2M (reduction ratio 62.0 ± 8.3%) and Cys (RR 73.2 ± 9.0%) than βTP (RR 26.3 ± 18.7%). Super high-flux membrane removed efficiently all the three LMWPs with a similar reduction ratio: β2M (RR 59.5 ± 16.2%), Cys (62.0 ± 7.5%), and βTP (53.4 ± 15.5%). The removal of the small molecules urea and creatinine was very similar with the different membranes, ranging 69.8–71.1% for urea, and 61.0–62.9% for creatinine.

## Discussion

In the setting of maintenance haemodialysis, a relationship between RRF and serum LMWP concentration has been indicated since long time [19, 20]. Different data indicate a different removal of the various LMWP, determined by the dimensions of the molecules and by the permeability characteristics of the dialysis membranes. In particular, a significantly lower elimination for βTP than Cys and β2M was found both in haemodiafiltration (HDF) and haemofiltration (HF). βTP was only moderately eliminated by HDF and not at all by HF, while standard haemodialysis (HD) with low-flux membranes did not remove any of the three LMWPs [21]. In the same period, another study found that the removal of βTP from the blood was modest and definitely lower than that of β2M after HD and HDF with high-flux dialyzers (KUf ranging 40–69 mL/h/mmHg) [22]. On the contrary, serum levels of βTP were tightly associated to residual diuresis of MHD patients suggesting that βTP serum levels may serve as a surrogate marker for RRF [20]. However, the possibility to evaluate RRF in dialysis patients from serum concentrations of the LMWP cystatin C has been addressed by different studies with conflicting results [23, 24]. No data is available on the effect of dialysis with super high-flux membranes on serum βTP levels.

Formulas based on serum levels of βTP and other LMWP measured before the dialysis session have been recently proposed to predict RRF in MHD patients avoiding urine collection [16, 17]. Some differences can be notices between the two studies. In particular, serum βTP was unaffected by haemodialytic treatment [17], while decreased after high-flux HD and even more after HDF [16]. The editorial comment to these papers proposes some caution notes due to the expected lower dialyzer clearance of βTP, whose MW is higher than β2M and Cys, and to the need for studies comparing βTP clearance with high-flux haemodialysis, superflux dialyzers, and high-volume haemodiafiltration [18].

Our previous results in CKD patients, not dialyzed, demonstrated that βTP is an adequate marker of GFR since its serum concentrations are determined exclusively by GFR and age (multiple correlation coefficient 0.9245) [15].

The present study, which aims to evaluate the effects of haemodialysis with low-flux, high-flux and super high-flux membranes on serum concentrations of ßTP in MHD patients with null RRF, was performed in reports data from a small number of patients, which is a limitation of the study. A strength of the study is the very wide range of permeabilities of the dialytic membranes from low- to super high-flux. No other data are available on the effect of dialysis with super-high flux membranes on serum levels of βTP in MHD patients. The study was undertaken in different patients for the different treatment strategies, which is another limitation of the study. However, dialyzer blood flow, dialysate flow, length and frequency of dialysis were similar in all patients, and blood samples were drawn 30 min after the end of dialysis to minimize eventual rebound phenomenon.

The results of this study demonstrate that the removal of βTP from blood is null with low-flux dialysis membranes, and progressively increases with the increase in the permeability of the membranes. Haemodialysis with super high-flux membrane has the highest efficiency in decreasing serum levels of βTP. The differences among βTP, β2M and Cys, observed during treatments with low- and high-flux membranes, become insignificant using a super high-flux membrane. Due to the different removal efficiency, higher serum βTP were found, before the first dialysis of the week, in patients treated with low- and high-flux membranes than in those treated by super high-flux membrane. These differences may have an impact on the values of residual renal function calculated by means of the recently proposed formulas [16, 17]. We could not evaluate the relevance of this effect in our patients, since they had no residual renal function.

## Conclusions

The plasma clearance of small proteins and particularly of βTP is dependent from the permeability of the dialysis membranes. Super high-flux membrane have the highest efficiency to remove ßTP from the blood. The differential elimination of small proteins in the different haemodialysis techniques may affect the reliability of the prediction of residual renal function from serum concentrations of small proteins.

## Abbreviations

CKD: Chronic kidney disease
Cys: Cystatin C
GFR: Glomerular filtration rate
LMWP: Low-molecular weight proteins
MHD: Maintenance haemodialysis
MW: Molecular weigth
RRF: Residual renal function
β2M: β2-microglobulin
βTP: β-trace protein
